# Learning from the implementation of Universal Free School Meals in Scotland using Normalisation Process Theory: Lessons for policymakers to engage multiple stakeholders

**DOI:** 10.1016/j.foodpol.2020.101936

**Published:** 2020-08

**Authors:** Stephanie Chambers, Nicola Boydell, Allison Ford, Douglas Eadie

**Affiliations:** a MRC/CSO Social and Public Health Sciences Unit, University of Glasgow, United Kingdom; b Usher Institute, Centre for Population Health Sciences, University of Edinburgh, United Kingdom; c Institute for Social Marketing and Health, University of Stirling, United Kingdom

**Keywords:** Schools, Food, Normalisation Process Theory, Policy, Universal, Meals

## Abstract

In 2014/15, Universal Free School Meals (UFSM) were introduced in Scotland and England for children in their first three years of primary school. This study examined the implementation of UFSM in Scotland using Normalisation Process Theory (NPT), a middle-range theory of implementation, to identify areas of learning for policymakers wishing to introduce or extend similar policies. NPT is predominantly used to evaluate interventions or new technologies in healthcare settings. Qualitative data were collected across Scotland using a case study approach shortly after implementation (n = 29 school-level stakeholders) and in the following school year (n = 18 school-level stakeholders and n = 19 local authority-level stakeholders). Observations of lunchtime in each school were conducted at both timepoints. Data were analysed using a thematic framework approach using NPT constructs and sub-constructs. Results suggested education and catering stakeholders experiences of implementation diverged most around the NPT concepts of coherence, cognitive participation, and reflexive monitoring. Lack of coherence around the purpose and long-term benefits of UFSM appeared to reduce education stakeholders’ willingness to engage with the policy beyond operational issues. In contrast, catering stakeholders identified a direct benefit to their everyday work and described receiving additional resources to deliver the policy. Overall, participants described an absence of monitoring data around the areas of greatest salience for education stakeholders. This study successfully used NPT to identify policy learning around school meals. Policymakers must increase the salience of such intersectoral policies for all relevant stakeholders involved before policy implementation, and plan adequate monitoring to evaluate potential long-term benefits.

## Highlights

• Universal free school meals (UFSM) could improve children’s outcomes.
• Evaluation of UFSM policies internationally is limited.
• UFSM policies need to engage with multiple stakeholders adequately.
• Policymakers must communicate the potential benefits to educational stakeholders.
• Adequate monitoring and evaluation could help to improve communication.

## 1 Introduction

### 1.1 Policy context

Within the United Kingdom (UK) and beyond, school meals are a long standing proposed solution to child malnutrition. In the 19th and 20th centuries the provision of food and/or milk within schools, either via charitable organisations or the state, were framed as a policy response to alleviating hunger and the conditions arising from poor nutrition (Harris, 1995, Hurt, 1985). In the 21st century, school meals have been viewed as a potential policy to reduce the likelihood of children experiencing overweight and obesity, particularly since the introduction of standards around the nutritional quality of foods/meals that can be served (Morgan and Sonnino, 2008). Since the financial crisis of 2008, and the subsequent policies of austerity in public sector spending, and widespread experience of wage deflation, school meals are once again being promoted as a solution to child hunger (Lambie‐Mumford and Sims, 2018). Around one in five children under 15 in the UK are estimated to live in households experiencing food insecurity (FAO, 2018, Trussel Trust, 2019) and the Trussel Trust have seen use of their foodbank network increase by 73% in the last five years (Trussel Trust, 2019).

Although policies to improve children’s health and wellbeing often receive high levels of public support (Chambers and Traill, 2011, NHS Health Scotland, 2017, Oliver and Lee, 2005), school meals have always been a highly politicised issue. In 19th and 20th century Britain, they were criticised as absolving parents of their responsibility to feed their children (Harris, 1995, Hurt, 1985). Means testing also resulted in families not taking up their entitlement to support, and there continues to be concern about the stigma associated with taking up a Free School Meal (Sahota et al., 2014, Woodward et al., 2015).

After the 2010 UK general election additional funding was provided for school meals as a result of the coalition deal between the Conservative Party and the Liberal Democrats (Liberal Democrats, 2010, Long, 2017). Scotland and England invested in Universal Free School Meals (UFSM) for children in their first three years of primary school, and the Scottish Government introduced UFSM within Scottish schools for children in primary school years 1–3 (P1-3) in January 2015.

At the 2017 general election, the Conservative Party included a manifesto commitment to remove funding for UFSM and invest instead in a universal breakfast programme, with an estimated saving of £4 billion per year (The Conservative and Unionist Party, 2017). The Labour Party campaigned for an extension of the programme to all primary school children, and continue to support this policy (Labour Party, 2017, Labour Party, 2019). Opinion polling at the time suggested that members of the public supported extending the policy to all primary school children (YouGov, 2017). Following the Conservatives formation of a minority government, this manifesto commitment was dropped, and UFSM continues for children in their first three years of primary school in England. Within Scotland, the governing Scottish National Party continue to support UFSM for P1-3 children, and have committed to provide free meals to all 2, 3 and 4 year olds who benefit from increased nursery provision by 2021.

With the potential for expansion of UFSM provision in the UK and beyond (currently full universal provision exists only in Sweden and Finland), it is important to revisit the implementation of the current arrangements to understand the potential opportunities for success, but also the potential for policy failure in the future. In this study we do this through an evaluation of UFSM, analysed through the lens of Normalisation Process Theory (NPT). NPT is a mid-range sociological theory that has been used to explore the work that organisations, and individuals within them, undertake to normalise and embed new initiatives/interventions into routine practice (O'Donnell et al., 2017).

### 1.2 Normalisation process theory (NPT)

NPT has been used to evaluate the processes involved in the introduction and implementation of health care interventions (May and Finch, 2009, May et al., 2009, McEvoy et al., 2014, Murray et al., 2010), but has not been used widely to evaluate the process of the introduction of wider healthy policy or population health interventions (see Segrott et al., 2017, Mackenzie et al., 2019 for exceptions). May and Finch (2009) define the normalisation process as,

the work that actors do as they engage with some ensemble of activities (that may include new or changed ways of thinking, acting and organizing) and by which means it becomes routinely embedded in the matrices of already existing, socially patterned, knowledge and practices. (p.540)

NPT consists of four main constructs (each with four sub-constructs) which describe the different types of work stakeholders engage in through the process of implementing and embedding a new intervention or policy. Coherence (sense-making) and Cognitive Participation (engagement) focus on the planning phase of an intervention, policy or programme, whilst Collective Action (enactment) and Reflexive Monitoring (appraisal) focus on the implementation phase (McEvoy et al., 2014). Table 1 provides an overview of the sub-constructs within NPT and their definitions.

**Table 1:** Overview of Normalisation Process Theory (NPT) constructs and sub-constructs.

Coherence (Sense-making)	Cognitive Participation (Engagement)	Collective Action (Enactment)	Reflexive Monitoring (Appraisal)
*Differentiation* Viewing policy as new way of working	*Initiation* Work of actors leading policy implementation	*Interactional workability* Range of interactions actors encounter in work to enable/hinder tasks	*Systematisation* Formal or informal collection of information
*Communal specification* Work undertaken to reach shared understanding of policy aims/outcomes	*Enrolment* (Re)organising others	*Skill set workability* Allocating work to appropriately skilled staff as policy implemented	*Communal appraisal* Actors’ collective evaluation of policy
*Individual specification* An actor’s understanding of tasks required to carry out policy	*Activation* Understanding practices required to sustain policy	*Relational integration* Confidence in new practices to sustain policy	*Individual appraisal* Individual actor’s understanding of how intervention affects them
*Internalisation* Perceived worth and benefits of engaging with policy	*Legitimation* Work to ensure actors recognise their role in policy implementation	*Contextual integration* Work shaped by resources and policies available	*Reconfiguration* Actors’ ability to change practices to improve policy outcomes

Wood (2017) has argued that NPT has substantial potential utility as a theory to understand why some interventions in education settings might be implemented, embedded and integrated (normalised) into every day practice, and why others may not. McEvoy et al. (2014) argue that an advantage to using NPT is that it can be used not only to understand past implementation, but also future implementation. This is a key strength when considering expansion of free school meals to a greater volume of pupils.

### 1.3 Aim

The aim of this study was to use normalisation process theory to understand the implementation of UFSM for children in their first three years of primary school within Scotland, and to use this understanding to identify key areas of learning for any further extension of the policy within the UK and beyond.

## 2 Method

### 2.1 Design

A qualitative case-study approach was adopted to collect in-depth information from a range of relevant stakeholders about their experiences of the implementation of UFSM in Scotland. The policy came into effect on 1st January 2015 and this research was carried out March–October 2015. At timepoint 1, data were collected in the months following implementation. At timepoint 2, data were collected in the new school year, with a new intake of primary 1 children. Ethical approval for the study was obtained from the University of Stirling’s Research Ethics Committee.

Across Scotland there are 32 local authorities with statutory responsibility for providing education and catering in over 2000 primary schools. We aimed to collect data from as wide a range of local authorities across Scotland as was possible within the constraints of the project. We identified nine local authorities that provided a range in terms of population density and levels of area deprivation. We selected three of these authorities to collect school-level data only, and six to collect local authority-level data. Selected schools and local authorities were considered case studies. Data were collected via in-depth interviews and observations within schools of lunchtime. An overview of recruitment is provided in Table 2.

**Table 2:** Sample overview.

Stakeholder level	Timepoint 1 (March – June 2015 post-implementation)	Timepoint 2 (September – October 2015 new school year)
**Schools (n = 3)** *School characteristics:* • 6 > 200 pupils • 6 in 40% most deprived datazones • Free School Meal uptake range 71%-99% • 3 in rural areas • 5 in highly urbanised areas	Lunchtime preparation & serving observations in 10 schools Interviews with: • leaders (n = 10) • head cooks (n = 9) • teachers (n = 10) • lunchtime supervisor (n = 1)	Repeat observations & interviews: • leaders (n = 10) • head cooks (n = 8)
**Local authorities (n = 6)** *LA characteristics:* • Deprivation levels^a^: 2 below 10%, 2 between 10 and 20%, 2 > 20% • Urban/rural classification: 2 predominantly urban; 3 mixed and 1 rural LA		Case studies in 6 selected local authorities. Telephone interviews with: • LA Catering (n = 11) • LA Education (n = 5) • Head teachers (n = 3)

a Deprivation levels defined as percentage of datazones within Local Authority boundary ranked in the 20% most deprived areas according to the Scottish Index of Multiple Deprivation.

### 2.2 School recruitment and procedure

Ten schools were recruited in the three school-level data only LAs. We recruited 3–4 schools within each LA as this provided the breadth to collect data from schools with different profiles within the limits of the project resources. School recruitment approaches varied by LA due to LA rules and preferences for the conduct of research studies within their jurisdiction. One LA sent information about the project to all schools within their area and invited them to contact the research team, with three schools (two of which shared a campus) subsequently doing so. We sent these schools the relevant project information sheets via email at that point and all three agreed to participate. In the other two LAs, schools meeting our criteria were identified via liaison between education and facilities management departments, who then invited the relevant schools to participate. Once schools had agreed to the research team contacting them, their details were forwarded to us, and we then made contact with them via email, sending the relevant participant information sheets. Our criteria were based on size, deprivation level of school postcode, and urban/rural level. School roll size ranged from 32 to 362. Five schools were located in older school buildings, whilst four were located in new buildings, including two schools who shared a campus and a dining space. Each school received a payment of £200 to cover the costs of staff participation in interviews.

School interviews were split into two timepoints in order to understand implementation in both the early stage (March – June 2015), and at the beginning of the following school year (August – September 2015). At timepoint 1, we interviewed school leaders (head and deputy head teachers) (n = 10), head cooks (n = 9), and P1-3 teachers (n = 10). An additional interview was carried out with a member of support staff who supervised the dining hall at the schools’ suggestion. These were key stakeholders within schools who the research team and project advisory group believed were likely to be involved in the implementation of UFSM and would have potentially divergent school-level perspectives. School leaders selected P1-3 teachers based on their availability and willingness to speak about the UFSM policy. At timepoint 2, we conducted a short interview with a senior leader in each school (n = 10). Additional informal interviews were carried out with eight of the nine head cooks interviewed at timepoint 1. Two observations of lunchtime preparations, serving and clean up were carried out within each school at timepoint 1, and a single observation at timepoint 2. Researchers made detailed field notes for each observation, and completed a structured observation pro forma for each school recording whether pre-ordering, cashless and queuing systems were in place, as well as use of the dining space, staggered servings, and lunchtime length.

Interview questions asked participants about: preparing for the implementation for UFSM (eg barriers and facilitators); experience of the implementation in the early stages (eg unintended consequences and mitigation of consequences); and of challenges encountered in the new school year (timepoint 2). Interviews lasted between 15 and 50 minutes. Participants provided written informed consent. All but two formal interviews were audio-recorded. A professional transcription company transcribed interviews and transcripts were checked for accuracy by the research team. Where audio-recording was not possible (for example, when head cooks were engaged in preparation and clean-up activities), detailed notes were taken instead.

### 2.3 Local authority recruitment and procedure

Data were collected at LA level from six LAs. These authorities were purposively sampled to ensure selection of a representative cross-section in terms of rurality, deprivation levels, types of catering provision and differences in level of uptake of UFSM in 2015 (Scottish Government, 2015). We wished to speak with both catering and education stakeholders within each LA to gain a range of perspectives of UFSM implementation, with an aim of speaking with two from each department. An initial list of catering and education leads was drawn up by members of the project advisory group as potential interview candidates. After making contact with these candidates, snowball sampling techniques were used to identify up to four stakeholders in each LA. Three LAs were unable to provide candidates from education to participate in the study, and therefore we interviewed a nominated head teacher to gain an education perspective. In one local authority the education department did not provide any support or guidance, therefore we recruited a head teacher independently using data provided by the local facilities manager. A total of 19 participants took part in an interview, 11 from catering, five from education and three with head teachers. Sixteen individual interviews were conducted by telephone using a semi-structured topic guide. Additionally, in one LA three catering representatives participated in a small group interview.

Participants were provided with an abbreviated version of the interview guide in advance of the interview. Interviews included the following topic areas: structure of school meals in LA; participant’s role; preparation for implementation of UFSM; feedback; barriers and facilitators to implementation; impact of policy; unintended consequences and policy learning. Interview length varied from 30 to 90 min. Participants’ provided informed consent. All interviews were audio-recorded and transcribed, again via a professional transcription company, with transcripts checked for accuracy by the research team.

### 2.4 Analysis

Transcripts from the school and local authority interviews were read and re-read by the research team. Broad inductive coding was originally carried out with extensive discussions about the similarities and differences across the school and LA levels. Data were then subject to coding using the NPT constructs discussed above as a coding frame in Nvivo 11. Codes were then examined via the different stakeholders interviewed (head cooks, school leaders, P1-3 representatives, LA catering representatives and LA education representatives). This allowed differences in approach by key groups of stakeholders to be identified. The research team engaged in continuous dialogue throughout the coding and interpretation process, challenging areas of uncertainty or confusion, particularly around the definition of NPT constructs, where necessary.

## 3 Results

To understand the implementation of UFSM, we present the results under the headings of NPT constructs. The results highlight where there were areas of overlap between different groups of stakeholders, but also emphasise key differences.

### 3.1 Coherence

#### 3.1.1 Differentiation

No participants described the introduction of UFSM as a completely new way of working. Multiple explanations were provided for this view, for example, school leaders stressed that all children had previously been accommodated in the dining hall, and therefore management of space, with or without introduction of UFSM, was an ongoing task. Few stakeholders described setting up new systems ahead of the implementation and instead continued with existing operational and engagement strategies.

In schools where a high proportion of children were eligible to receive FSM under the previous means tested system, leaders did not perceive that the policy would lead to a substantial increase in volume of meals served.

*We were dealing with a high number of children already who were receiving free school meals.* (School Senior Leader, School 6)

Nevertheless, catering staff at school and LA level noted that they expected UFSM would increase the volume of meals served and this would likely result in changes to their way of working. Staff recognised, and articulated, a need for a more actively managed way of working not only to deal with increased demand, but also uncertainty within the initial implementation period.

#### 3.1.2 Communal specification

LA catering staff described meeting with school head cooks and leaders ahead of policy implementation. Head cooks and school leaders also met separately. In describing these meetings these three groups of stakeholders focused primarily on the need to make the policy work. The interpretation of this was operational - ensuring that all children were adequately fed in the time available, improving and upgrading facilities and equipment, and that training was in place.

*We tried to sort out the operational challenges, briefed the staff, and we’re very good in schools at making things work because we have to. These wee people need fed.* (School Senior Leader, School 5)

Stakeholders did not describe these meetings as including discussions around the wider long-term aims of the policy, the long-term potential benefits, or the likelihood of achieving these.

#### 3.1.3 Individual specification

Nearly all stakeholders interviewed outlined understanding of some of the key tasks that the policy’s introduction would require of them individually. These again were largely operational, for example, ensuring that all children could be fed within the time allocated. Teachers and local authority education representatives did not discuss having extensively reflected on whether their everyday tasks would change in relation to UFSM’s introduction. Local authority catering representatives however reported that they supported schools ahead of the implementation by carrying out visits and audits of facilities, equipment and staffing levels.

*Where we could foresee there would be challenges within the school. So lack of space or not enough tables and what have you. We started to go round those head teachers before that and agreed trial dates for their particular school*. (LA Catering Rep, LA 2)

Head cooks said they had reflected on pressure points within the lunch system in their schools, such as complicated menus, high volume days and at clear up.

*We had a couple of trial runs on busy days, theme days. Where instead of doing 110 customers a day we were doing 240–250… So, we knew what we were going to be coming into, we knew the numbers, and we coped.* (Head Cook, School 1)

A key task identified by both school leaders and local authority catering representatives as being of particular importance was the need to communicate with parents about the changes to school meals.

#### 3.1.4 Internalisation

The most striking aspects of Coherence identified for the implementation of UFSM was internalisation, that is the work undertaken to understand the potential benefits of an intervention or policy. No one group of stakeholders had a homogeneous view on the purpose or value of the policy, and it was in this area groups of stakeholders appeared to diverge most in their understandings of the potential benefits. The results suggest substantial confusion around why the policy had been implemented, and what the main outcomes were that the policy hoped to achieve. It was also clear that the policy was viewed as being politically driven, with some stakeholders responding as both an individual citizen to it, as well as a professional involved in the implementation of it. Meetings set up ahead of implementation had not appeared to focus on discussion of the potential benefits or the value of UFSM.

The main themes that were discussed in relation to the value or benefits of UFSM were questioning the appropriate use of public money, the potential for families to benefit financially, health and social benefits, and sustaining the school meal system.

Some senior school leaders, local authority education representatives and teachers questioned the introduction of a universal benefit, such as UFSM, given a financial climate in which they were facing substantial cuts to education budgets. They argued that under a means tested system the most vulnerable families were already benefitting, with limited perceived stigma, and that affluent families were now receiving unnecessary government support that could be invested in reversing cuts to teaching and support staff numbers.

*Some of our Head Teachers, what they’re saying really is that…this sort of universal benefit, for instance, actually it’s not needed, you know, because most of the parents in our local authority can well afford to pay.* (LA education rep, LA 5)

Some school leaders made the distinction between supporting the principle of a universal system that encouraged equality amongst children with the practical reality of running a school under severe financial pressure. Stakeholders, including some local authority catering representatives, perceived the policy to be a ‘vote-winner’, and cited this as its main rationale.

*We reckon [UFSM] was a vote catcher [Laughter]. Maybe I’m a bit too cynical in ma old age. It was very political. It was a vote catcher.* (LA education rep, LA 4)

Nevertheless, all groups of stakeholders recognised that there were a number of potential benefits to the policy. A key one was that families who previously had been ineligible for assistance could now receive a meal for free, e.g. those working but living on low incomes. School teaching staff and leaders particularly highlighted this as a policy benefit.

*We do have pockets of deprivation and those children I suppose in the past would have qualified for a free school meal. But then, we always felt there were one or two that maybe were just over and didn't qualify.* (P1-3 Teacher, School 9)

Another perceived benefit was the perception that school-provided meals were of a higher quality than lunches provided from home. Stakeholders, particularly LA catering representatives, argued that school meals helped establish healthier eating habits and exposed children to a greater variety of foods, leading to nutritional benefits, and viewed hot meals of substantially greater benefit to children than packed lunches. Stakeholders recognised a social benefit to all children sitting together eating the same meal, and head cooks and local authority catering and education representatives said they believed the policy would help boost children’s school performance.

*I would hope that if the children are better fed at lunch time, that their learning experience is better in the afternoons an’ that that’ll have a positive effect on their attainment.* (LA Catering Rep, LA 1)

Interestingly, this was not a benefit discussed by school leaders or teachers who were the two main groups of stakeholders who questioned the nutritional quality of meals provided.

Finally, head cooks and LA catering representatives discussed a perceived benefit of the policy ensuring the sustainability of the school meal system. They noted that there had been a substantial financial gain to the catering departments of local authorities through the allocation of funding from the Scottish Government which allowed facilities and equipment to be upgraded. These stakeholders reported that funding had created and secured jobs, whilst the provision of a free meal engaged more children in the school lunch system early, with the hope being that they would remain as paying customers in the later years of primary school.

### 3.2 Cognitive participation

#### 3.2.1 Initiation

LA catering representatives were the main group of stakeholders who described leading initiatives to engage others in UFSM implementation. In liaising with school head cooks and school leaders, they attempted to engage these groups in the planning and eventual delivery of the intervention. A recurring theme that emerged across the interviews was that the separation between education and catering hampered the process of planning for implementation. LA catering representatives discussed tensions between their teams and school leaders and a lack of engagement from school leaders, particularly around increasing uptake of UFSM.

*Just a complete lack of co-operation. Complete lack. [School leaders] find it very time-consuming, they don’t find it to be - they see no worth in it - so therefore they fail to buy into it and support [catering] in trying to maximise the numbers.* (LA Catering Rep, LA1)

The explanation provided for this lack of cognitive participation included not seeing a benefit to their work, as exemplified in the quotation above, but also a reported belief that education colleagues were overloaded, social and physical distances within school buildings between catering and education contributed to siloed ways of working, and the organisational and financial structures within local authorities.

LA Catering representatives wished to engage more directly with parents to encourage uptake, but argued that opportunities were limited by schools. LA catering representatives also described a wish to engage teachers further, as they felt teachers could provide vital support in the dining hall. In one LA where the catering representatives had met enthusiasm within some schools, they asked school staff to share good practice with other schools in their local area (at joint meetings) in an attempt to engage them in the policy.

#### 3.2.2 Enrolment

Enrolment is closely related to initiation, but focuses more on the reorganisation that ensures that key groups take forward the work needed to successfully implement the policy. In the case of UFSM, this involved some higher level discussions between the local authority catering team and school senior leaders (as highlighted as part of the initiation process), however, there was greater discussion around efforts within schools to enrol key individuals and groups ahead of the policy’s implementation. For example, a head cook in one school described building relationships with dining hall supervisors to identify children not eating enough at lunchtime. School senior leaders described discussing UFSM with head cooks and other school catering staff to identify how changes could be made to ensure the smooth running of the policy.

*Part of that process is working with my catering colleagues, you know? I think I work quite closely with them, I try to build relationships there so that we can work together in the best way possible really. I see that as part of my role is to make sure that people are working together. So, as well as overseeing the systems, it’s about making sure that people collaborate and work together.* (School School Senior Leader, School 1)

As previously discussed, school senior leaders described an ethos of ‘making things work’, and therefore articulated feeling responsible for the operational implementation of UFSM.

#### 3.2.3 Activation

Stakeholders described undertaking a variety of tasks to sustain the intervention. Despite the view from LA catering representatives that school senior leaders were not sufficiently engaged in the implementation of the policy, the work described by these school staff suggested that they were involved in a continual process of active management of lunchtime routines. They discussed the need to ensure a positive dining experience (as did local authority representatives). This was achieved by school senior leaders being present in the dining hall, providing practical support to children, identifying pressure points, asking P1-3 teachers to supervise, and implementing a buddy system with older children supporting younger ones. In a number of schools, work had been undertaken to change the timings of lunch to ensure all children could be served. Other work carried out at a school level by school teachers and senior leaders was identifying and engaging with families that they perceived would benefit most from UFSM to encourage them to take up the meal being offered.

LA catering representatives were also involved in activities to sustain the intervention. These included altering menus when necessary to increase their popularity or to reduce preparation time; overseeing work to upgrade to kitchen/dining facilities and equipment; arranging with head cooks taster sessions for parents and children; providing photographs of menu items to display in schools to help children make choices; and in some local authorities implementing pre-order and/or cashless systems.

#### 3.2.4 Legitimation

Legitimation focuses on a stakeholder’s belief that it is appropriate for them to be involved in the implementation of an intervention. The main area of tension identified around this area was the extent to which education staff at all levels were actively involved in implementation. Indeed, whilst the ethos of ‘making things work’ helped to ensure the policy could be sustained within schools, the implication was that schools were not involved in driving forward UFSM.

*UFSM did implement quite smoothly, with no issues. You could argue that if you were planning it again, you would have spent more time on each of the sites, speaking with the local, the Senior Management Teams, Education teams on the sites, to say, ‘This is what we’re gonna be looking at. This is what’s gonna happen, potentially. How do you want it to work on this site?’ But actually, I’m not gonna say by default, because actually it worked, but by default it worked.* (LA Catering Rep, LA 3)

School senior leaders discussed having a role within delivery of UFSM, however, it was clear that this related to active management, rather than active leadership.

*The catering department, they organise everything, and my role really is just to fit into that system and I would say is, oversee systems and procedures and just check that it’s working well. Sometimes it isn’t but it’s things that are out my hands.* (School Senior Leader, School 1)

Although the time school senior leaders spent in the dining hall was described as important in ensuring lunchtime operated smoothly, a number of them highlighted that their main rationale for being present was to interact with the children. They also stressed that they desired greater recognition for the time that they and other education staff spent in supervising lunchtime.

### 3.3 Collective action

#### 3.3.1 Interactional workability

Participants discussed the ways in which the work they undertook as part of implementing UFSM interacted with other tasks. There were few areas reported where UFSM made accomplishing tasks easier. No longer having to collect cash from children was one of the few ways that teacher and support staff administrative time was reduced. Nevertheless, other schools reported that teacher and support staff administrative time had increased through facilitating pre-ordering systems and supervising children in dining spaces. For head cooks, the policy’s implementation required extra time for preparation and clear up, storage of food had become more problematic, paperwork had increased, and some menus could no longer be delivered.

*It's at the end o' the day when the kids have all had their lunch an' you're left wi' dishes stacked sky high. That's where it came in more for us than anything…And the added paperwork.* (Head Cook, School 7)

A number and range of stakeholders discussed UFSM making it more difficult to meet Scottish Government directives on delivered hours of Physical Education each week as many dining spaces were also required for this purpose. There was concern that the policy undermined the children’s dining experience, with insufficient supervision provided, increased queuing and more noise. Some participants also expressed concern about the policy increasing food waste, which they aimed to keep as low as possible. Finally, although there was acknowledgement that UFSM meant that there was less opportunity for children to be stigmatised, a small number of participants reported that it was now more difficult to identify eligibility for other means tested benefits such as clothing allowance for school uniforms and free milk.

#### 3.3.2 Skill set workability and relational integration

The key points raised under these concepts overlapped and were discussed in a somewhat limited capacity and are therefore presented together. Skill set workability, the allocation of work related to UFSM, was dependent on having staff who were adequately trained and prepared to carry out the work necessary (relational integration). LA catering representatives appeared to have confidence in the skills of catering staff working in schools as the policy was implemented. Some had provided additional training to existing staff for new equipment and preparation processes. Extensive recruitment of catering staff had also taken place. For some local authorities, this recruitment had been impeded due to lack of lead-in time and the policy implementation coinciding with the Christmas period. LA catering representatives and head cooks discussed the need for staff flexibility within this environment to ensure that all tasks could be completed. In some schools, the relationship between education and catering staff was raised as an issue potentially undermining more widespread uptake of school meals, as evidenced earlier. Different stakeholders also raised concern about lack of supervision of children in the dining hall, noting that failing to support younger children at lunch could serve to undermine the policy as children could become unfocused and thus less likely to eat the food on offer. The majority of participants who raised this as an issue felt that training of dining hall supervisors would be helpful.

*But [supervision] is where [catering would] like to say, ‘What schools need a wee bit of extra help in the dining room? Can we employ extra people just solely to go out in the dining room and assist with that process?’ It would help schools and our staff. It would help build bridges.* (LA Catering Rep, LA 2)

A school senior leader and a teacher said they were concerned that poor communication between catering staff and young children also undermined the policy by contributing to a poorer dining experience. Nevertheless, other school-based education staff praised catering staff communication with the children.

Some LA catering representatives reported that the implementation had resulted in fewer challenges than they had expected. The majority of the participants reported that they had confidence in the way in which the policy was working. Some school senior leaders felt that with their active management of lunch, the policy had been implemented successfully, whereas others commented that queuing was an ongoing issue. Some also raised concern about the capacity for the dining hall to meet demand in the longer term as school rolls increased.

#### 3.3.3 Contextual integration

The allocation of appropriate resources was crucial to the successful implementation of the policy from catering stakeholders’ perspectives. The most obvious allocation of resources came via the Scottish Government in the form of payments to local authorities. There were payments to upgrade facilities, but also payments based on a projected uptake amongst P1-3 pupils. The increase in budgets for local authority catering departments allowed them to hire more staff, increase staff hours, pay overtime for staff training, upgrade facilities, and buy new equipment. Although catering staff were enthusiastic about increased financial resources, they were critical of the late notification of capital funding which had delayed some of their upgrading work. Only two stakeholders from education discussed additional funding as being a resource that they could draw from in implementing the programme. Indeed, there were complaints that widespread additional funding for administration or supervision had not been provided. This aligned with the perception that education budgets were being slashed at the same time, creating a feeling of competition around resource allocation.

*That’s why I get annoyed about Free School Meals because our support staff budget is reduced but they’re giving kids Free School Meals.* (School Senior Leader, School 1)

Schools were also concerned about the long-term viability of delivering the policy successfully with increasing school rolls, citing the additional strain on dining facilities where these had not been upgraded or expanded.

Other resources drawn upon have been discussed in previous sections but included catering staff (at school and local authority-level) being able to draw support from school staff, including help from older pupils. Resources included the perceived willingness of school senior leaders to make operational changes to meet increased demand, as well as school staff acting in a supervisory capacity in the dining hall. This supervision was greatly valued by LA catering representatives and several said they believed that this was an area that required further investment, as it was the best way of improving the dining experience for children. A small number of senior leaders and teaching staff noted that lunch was protected time for teachers as part of their work contract, and as such, there was no obligation or expectation upon them to provide this supervision.

Other resources provided by the local authority that were highlighted as being particularly important to head cooks were the redesign of menus to allow for quicker preparation on days where high volumes of children were expected to be processed through the dining hall; changing delivery arrangements to reduce pressure on storage facilities; tailoring menus to individual school circumstances; and LAs supporting cooks to introduce more taster sessions.

### 3.4 Reflexive monitoring

#### 3.4.1 Systematisation

With the exception of uptake, there were a lack of formal mechanisms to support the systematic collection of data on outcomes in relation to the success of UFSM. Records on uptake were generally kept meticulously by head cooks, and this information was returned to local authority catering departments. Catering-related staff were therefore generally able to report uptake across the local authority using these figures. It was clear however that there was a lack of data collected around other potential outcomes of the policy, such as parent and child experiences of UFSM. Furthermore, at the time of the interviews, there did not appear to be any long-term plans to assess whether UFSM had provided an increased nutritional benefit, contributed to reducing stigma or improved children’s school performance. Instead, appraisal was generally anecdotal in nature.

*I’m not aware of there being any way that we can feedback [to the local authority] what we know and what we see to them…I don’t think they’d do anything about it anyway, because—it’s a bit like everything else. Somebody that doesn’t actually do your job makes your decisions for you and you’ve just got to do it.* (P1-3 Teacher, School 7)

#### 3.4.2 Communal and individual appraisal

Participants were asked to consider whether USFM had been implemented successfully. There was limited discussion of different stakeholders coming together to assess whether the policy was working well. Some head cooks and school senior leaders described meeting to discuss how the policy was working, as well as some head cooks discussing this within the teams they led. There appeared to be limited communal appraisal between local authority catering representatives and education-related staff, reflective of competing priorities in day-to-day tasks.

In terms of individual appraisal, the success of the intervention was judged by head cooks and LA catering representatives mainly based on uptake figures and in some cases also changes in levels of food waste. The majority were keen to increase P1-3 uptake to as high a level as possible.

*Last week was our first week of the Primary 1s being full-time, so our uptake last week was 70%. And that’s Primaries 1 to 3, vis-a-vis the numbers in the Primaries 1 to 3.* (LA Catering Rep, LA 1)

In one LA, however, they did not want to increase uptake beyond current levels as they reported that the Scottish Government would only reimburse at a level of 75%, and therefore, the LA would have to subsidise above that level. Reported uptake levels were variable when compared across local authorities, but also within local authorities. Various explanations were put forward to explain differences in the levels of uptake within, and across schools. These included levels of affluence/deprivation, fussy children, menu choices, attitudes of education staff, and perceptions of the dining experience.

School senior leaders and P1-3 teachers focused less on uptake, and appeared to judge successful implementation of the policy operationally, i.e. whether all children in the school could be fed during the time available for lunch. This was also important to head cooks and LA catering representatives. Additional areas that were put forward as evidence for success or otherwise were the perception of the impact of UFSM on children’s dining experience (noise, increased queuing); the quality and perceived nutritional value of the food available; food waste; and some additional vulnerable families benefitting from the policy.

#### 3.4.3 Reconfiguration

As the UFSM policy places a statutory duty on local authorities, stakeholders were limited in the ways in which they could make changes to the policy itself. Nevertheless, there were smaller-scale changes in terms of implementation discussed by both catering and education stakeholders. For education stakeholders, evidence for reconfiguration was generally based on experiential learning, whilst catering stakeholders also drew on systematically collected data, as detailed previously.

Changes made by local authority catering stakeholders included increasing and monitoring catering staff ratios within schools and changing menus to make serving large numbers of children more efficient.

*In some schools, because the uptake is so high, we have gone to one choice of hot meal…to make it quicker to serve. Schools with big school rolls and smaller dining rooms.* (LA Catering Rep, LA 4)

After implementation, education stakeholders (often in discussion with school-based catering staff) were involved in making further changes to the structure of lunchtimes in response to long queues, too few seats being available, and too little time for children to finish meals. By extending the length of lunchtime, changing rotas and managing the space available, they were able to mitigate unintended consequences. A small number of education stakeholders said that they had ongoing concerns around these issues.

*We just spoke to [school support staff], because obviously with changes, any changes, like, we’re speaking to them. “How’s it going? What are you finding?”…They’re saying “No. It’s too big queues, [children are] having to wait too long. They’re still not served, the bell’s ringing, so…” “Well what do you think? What will we try?” Say “We’ll try that. If it’s not working, we’ll try something else.”* (School Senior Leader, School 9)

Only catering stakeholders, particularly at LA level, outlined longer-term aims in relation to reconfiguring UFSM. For most, this related to increasing uptake, enhancing the sustainability of the school meals service.

*We are doing kind of surveys of the different kinds of stakeholder groups so school management, parents, councils and pupils, to look at, and that is not just primary school we are doing that across all sectors to look at you know, what is good, what is bad, what they like, what they don’t like, what would encourage them to take meals.* (LA Catering Rep, LA 4)

They also voiced a strong desire to improve children’s dining experience and described ways in which this might be possible by using additional funding to improve facilities and support high quality supervision within the dining hall. Education stakeholders did not discuss any longer term aims in relation to the ongoing implementation of UFSM.

## 4 Discussion

### 4.1 Consideration of findings

The findings highlight a number of areas of learning for policymakers should they wish to expand UFSM further, or if a similar policy were to be implemented in another jurisdiction. There are currently only two countries (Sweden and Finland) that offer UFSM to all children, however, researchers in numerous countries are debating how they might change their systems to improve children’s outcomes (Gaddis and Coplen, 2018, Gordon and Ruffini, 2018, Hernandez et al., 2018, Lucas et al., 2017).

These main areas of learning relate to coherent understanding of the purpose and potential benefits of UFSM amongst the stakeholder groups involved in its implementation, and monitoring. The policy’s long-term purpose was not discussed explicitly by the relevant stakeholders. Preparatory meetings focused more on the operational work to deliver the policy, rather than explicit discussion of the aims, purpose and potential longer-term outcomes. The perception of the policy as highly politicised appeared to create resentment toward UFSM, particularly by school senior leaders. Catering staff at both school and LA levels were able to see direct benefits for their own jobs stemming from the policy’s introduction, which perhaps further normalised the policy into their practices. School senior staff were less likely to discuss the policy of being directly beneficial to their job. In 2014, an evaluability assessment of UFSM was carried out with Scottish Government policymakers, with a theory of change developed (Beaton et al., 2014). Policymakers and researchers identified the longer term purpose and benefits of UFSM as being: cost savings for families; improving the healthfulness of children’s diets, leading to child healthier weight; and better school attendance and behaviour resulting in improved educational attainment.

The gap between education staff’s ‘sense-making’ about the policy and those of policymakers appeared to impact on other areas of work (such as cognitive participation and collective action) around UFSM’s implementation. LA catering representatives felt that many education staff were unwilling to engage with them to deliver the policy to the highest standards possible. Indeed, although education staff discussed an ethos of ‘making things work’, and therefore a commitment to delivering the policy, this did not appear to translate into taking a lead or necessarily achieving longer term benefits of UFSM, such as nutritional benefits or improving school performance. Lack of recognition of the time educational staff provided to support UFSM by LA catering departments, particularly in terms of funding, also appeared to undermine the extent to which education staff believed they had a legitimate role within the delivery of UFSM.

The findings presented on collective action further highlight why lack of buy-in from education staff might be problematic for the policy. It was clear that UFSM made very few tasks easier for education staff, which threatens to further undermine long term buy-in to any extension of the policy. It was evident also from interviews with catering staff how important adequate funding had been for them to implement the policy, and indeed, had helped to increase the coherence of the policy for them. Instead, education staff were provided with few extra resources, and there appeared to be an unspoken reliance on their willingness to make the policy work without financial compensation.

Finally, the findings on reflexive monitoring indicate that formal data were only rigorously collected on uptake. This is problematic as uptake is essentially an intermediary outcome, rather than a long term policy aim, as identified in the theory of change during the evaluability assessment (Beaton et al., 2014). Although catering staff were keen to focus on this outcome due to its relevance to their day-to-day role, it was of less relevance to education staff. Where these staff voiced support for the policy, it was in relation to nutritional benefits for children, reducing inequality and benefitting families. This suggests that there is a need to collect data systematically to measure these kind of outcomes, or use existing data sources that can provide measures of policy effectiveness (Beaton et al., 2014). Stakeholders repeatedly highlighted that there were few attempts to gain feedback on UFSM from parents or children, the groups that the policy is supposed to benefit most. Interestingly, when discussing issues related to coherence, few education staff said that they expected the policy to improve educational performance and/or attainment. It was instead LA catering representatives who identified this as a likely benefit of the policy.

The issues raised suggest that there are problems that need to be addressed before further extensions of the policy are implemented. The impression that education stakeholders appeared less invested in UFSM than catering stakeholders was evidenced further by the fact that education staff were involved in delivering the policy, but received little extra financial resource to enable this. Indeed, given the cuts that school senior leaders described experiencing within their budgets, a number expressed open resentment about the large-scale funding of UFSM, whilst they perceived that children’s educational experience had suffered. Without their buy-in however it is unlikely that the potential health benefits of the policy will be realised over time.

Wood (2017) highlights as a key barrier to change within educational contexts “policy and strategy overloads” that result in staff having too little time available to engage fully in significant change. In line with our findings, he argues,

*“The focus on coherence at the start of a change process ensures that individuals have a genuine and meaningful opportunity to discuss how a new practice is understood, what it is hoping to achieve, and what the benefits might be in adopting it. This helps to instil a greater sense of agency across the organization, and locates the change process within the team rather than positioning teachers as mere participants in someone else’s project”* (Wood 2017: 37)

### 4.2 Policy implications

The results of this work suggest that future long-term success of an extension of Free School Meals to either younger or older children, or in other jurisdictions, requires greater attention by policymakers to the process of sense-making and cognitive participation for those key stakeholders involved, particularly educational stakeholders. Japan is an exemplar country where this more integrated approach has been implemented, although the system is not fully universal with parents contributing to the cost of food. In Japan the Diet and Nutrition Teacher System is in place to support the delivery of school lunch, but also to provide pedagogical instruction within schools around diet and nutrition (Tanaka and Miyoshi, 2012).

We identified three ways that greater integration could be achieved within the UK. The first is to ensure that education also receives financial resource to implement the policy or extensions to it, for example, through funding adequately trained supervisory staff in the dining hall. The second is to collect and analyse data on outcomes that are meaningful to education stakeholders. These outcomes include the benefit to families, nutritional benefits and school performance. It was notable that none of the stakeholders described any formalised attempts to gain feedback from children and families specifically on UFSM. Some limited evaluation work has been carried out with parents around UFSM in Scotland suggesting that they welcomed and supported the policy, and were pleased with its potential to eliminate the stigma that surrounds a means-tested system (Ford et al., 2015). The third way to support the policy is to prioritise the need for strong communication at all levels between catering and education colleagues, particularly around the cognitive participation concepts of initiation, enrolment and legitimation. This could include local authority education staff being key stakeholders in meetings around planning, designing and monitoring the policy. At school level, policy implementation seemed to be most straightforward in schools where the relationships between catering and education staff were positive and open. In these schools, head cooks and school senior leaders met ahead of policy implementation to determine the ways in which it would work best within their contexts, and revisited arrangements after implementation and in the new school year, making changes where required. These findings underline the importance of establishing partnerships at school level as part of policy development, and including schools with different relationships and organisational structures in any pilot work.

Previous research evaluating UFSM in other contexts are relatively limited (Oostindjer et al., 2017). Countries like Sweden and Finland provide free meals to all school children, but it is methodologically challenging to evaluate policies that have been embedded for many years in an effort to demonstrate the benefits of a universal approach. Pilot schemes have been evaluated in both Scotland and England previously, however, these evaluations have focused on relatively short term issues and outcomes (MacLardie et al., 2008, Rahim et al., 2012). In early 2018, an evaluation of UFSM in England was conducted on behalf of the Lead Association for Catering in Education (Sellen et al., 2018). Results suggested that uptake was higher than that of Scotland. Qualitative research with school leaders suggested that there was some limited recognition of UFSM as coinciding with improvements in school performance, but that these staff were reluctant to attribute this to UFSM specifically, rather than wider ranging school food policy changes. Similar to our study, some school leaders reported that the introduction of UFSM had resulted in additional senior and teaching staff time spent on catering-related issues. Further work is necessary to determine whether staff such as these faced a similar sense-making and implementation process as education staff in Scottish schools, particularly as many English schools have a direct relationship with a caterer, rather than through a local authority.

### 4.3 Normalisation process theory and understanding policy implementation

As far as we aware, NPT has not been used previously to understand food policy, however, this study has benefited substantially by its application in the case of UFSM implementation. Using the NPT framework we have been able to systematically and theoretically investigate the implementation work undertaken by a range of stakeholders involved in the delivery of UFSM. A main advantage of applying the NPT framework was that the identification of evidence for each of the sub-constructs within the data aided understanding of the more subtle nuances within each of the four main constructs. For example, within cognitive participation, we were able to identify that education stakeholders were undoubtedly involved in the planning stage, through activation, but were less involved in driving forward the policy and engaging others in it. The conceptualisation of each of the four main constructs as phases was also helpful in considering how the policy progressed over the year, and leant itself well to the longitudinal elements of the data where school-level stakeholders were followed up. This was especially true of the data presented on Reflexive Monitoring, where we examined how schools had reflected on the normalisation of the policy once implementation was under way, and particularly in the new school year. We are aware however, that to treat the NPT framework as a strictly linear one, oversimplifies it. Undoubtedly, there is potential to move back and forward between phases as policies are embedded, and indeed we argue that this is necessary in the case of UFSM, as education stakeholders must be engaged more in making sense of the policy if it is to be expanded successfully. We believe that this is a process that will take longer for these stakeholders to meaningfully engage with, and is reliant on the collection of data that demonstrates the potential benefits of UFSM to those stakeholders.

The application of NPT to UFSM also furthers understanding of the utility of NPT beyond healthcare in examining wider policy implementation. It was undoubtedly challenging to ‘translate’ some of the concepts and subdomains to apply to a policy rather than a health intervention. We were aided in this task through the work of McNaughton et al. (2020), who have ‘translated’ the concepts for application to qualitative data, which simplified some of the descriptions into less technical terminology, allowing for a clearer application to an area of policy. Nevertheless, we found some difficulties in separating out individual and collective activities at times, and found there were extensive evidence for some subconstructs (eg internalisation), but less for others (relational integration). We concur with Wood (2017) that NPT is a useful framework for retrospectively examining the process of implementing educational policies and interventions, but would also be useful during the process of developing policies and interventions and anticipating issues that may act as barriers prior to implementation.

### 4.4 Strengths and limitations

A main strength of this work is that it provides one of the few academic studies of implementation of universal free school meal provision. Without this kind of research, there is no evidence base on which to underpin future policy in this area nationally or internationally. This is an area that is highly policy relevant. In 2018, the UK government published an update to their childhood obesity strategy (Department of Health and Social Care, 2018). They reiterated that school meals are an area that can contribute to improving children’s long-term health. The Scottish Government have similarly recognised this in their obesity strategy, and there continues to be substantial policy focus on reducing health and wider inequalities (Scottish Government, 2018).

A further strength within this study was our inclusion of a wide range of stakeholders. This allowed us to identify distinct differences in the response to the policy based on the role of the stakeholders involved. The study would have benefited from greater representation of local authority level education stakeholders, however, we were unable to recruit participants from this grouping in three of the six local authorities we were collecting local authority-level data from, and the views of senior school leaders substituted in these areas.

Both a strength and limitation of this work is its focus on the Scottish context. Whilst UFSM in Scotland and England has been implemented similarly, there are likely to be contextual differences that need to be taken into account in applying any policy learning across the UK and beyond. A further limitation is that whilst we are critical that schools and local authorities had not sought the views of parents and children on UFSM, the current study also suffers from their absence. This deficit of views from end users has been criticised in NPT studies previously (McEvoy et al., 2014). More engagement with these two key stakeholder groups is required in future work.

### 4.5 Conclusion

Interviews with key stakeholders delivering UFSM in Scotland highlighted that they were able to implement the policy as required, but that key areas need to be addressed if universal free school meal policies are to be extended or rolled out in other jurisdictions. This study has shown that the differences in opinion and approach of catering and education stakeholders must be addressed if there is to be a wider roll out of universal provision of free school meals in schools. By doing this, there is likely to be greater buy-in for all involved in delivery. Greater focus on the longer term aims of these types of policies is also essential through robust evaluation and high quality communication between all stakeholders involved.

## CRediT authorship contribution statement

**Stephanie Chambers:** Conceptualization, Methodology, Investigation, Formal analysis, Writing - original draft, Funding acquisition. **Nicola Boydell:** Investigation, Formal analysis, Writing - review & editing. **Allison Ford:** Methodology, Investigation, Formal analysis, Writing - review & editing. **Douglas Eadie:** Conceptualization, Methodology, Investigation, Formal analysis, Writing - review & editing, Funding acquisition.
